# *Ginkgo biloba* Efficacy in the Treatment of Drug-Induced Parkinsonism: A Randomized Clinical Trial

**DOI:** 10.5812/ijpr-134722

**Published:** 2023-07-31

**Authors:** Niayesh Mohebbi, Arash Kalantar Mehrjardi, Maryam Mousavi, Maryam Taghizadeh-Ghehi, Mahya Rezaie, Zahra Hooshyari, Faezeh Gholamian, Fatemeh Mohammadian

**Affiliations:** 1 Department of Clinical Pharmacy, School of Pharmacy, Tehran University of Medical Sciences, Tehran, Iran; 2 Research Centre for Rational Use of Drugs, School of Pharmacy, Tehran University of Medical Sciences, Tehran, Iran; 3 Research Center for Rational Use of Drugs, Tehran University of Medical Sciences, Tehran, Iran; 4 Faculty of Psychology and Educational Science, University of Tehran, Tehran, Iran; 5 Department of Psychiatry, Roozbeh Hospital, Tehran University of Medical Sciences, Tehran, Iran; 6 Department of Neurology, Roozbeh Hospital, Tehran University of Medical Sciences, Tehran, Iran

**Keywords:** Psychiatric Patients, *Ginkgo biloba*, Drug-Induced Parkinsonism

## Abstract

**Background:**

Drug-induced parkinsonism (DIP) is one of the most common movement disorders in approximately 20 - 35% of patients on antipsychotic medications. Managing the symptoms of DIP is challenging due to the limited number of potentially effective medications. On the other hand, this restricted possible treatment could have numerous side effects that ultimately result in patients stopping the medication all at once. The neuroprotective property of *Ginkgo biloba* extract (EGb) emerged as an effective commodity for the additional treatment of psychiatric disorders.

**Objectives:**

This study aimed to evaluate the efficacy of EGb in psychiatric patients with symptoms of DIP.

**Methods:**

A sample of 63 patients who met the inclusion criteria were recruited and randomly assigned to control and experimental groups. Both groups were followed for 3 months. One group received 80 mg of *G. biloba* three times a day, and the control group received a placebo. The patients were evaluated using the Unified Parkinson’s Disease Rating Scale and Montreal Cognitive Assessment.

**Results:**

*Ginkgo* could change the intensity of rest tremors, the severity of motor symptoms, rigidity, and bradykinesia. *Ginkgo biloba* might alleviate the severity of parkinsonism and motor symptoms and could lead to changes in the two components of working memory and short-term memory.

**Conclusions:**

*Ginkgo biloba* extract can be used as an effective and safe treatment in the management of DIP, whether in patients diagnosed with psychotic disorders or mood disorders.

## 1. Background

Drug-induced movement disorders, including drug-induced parkinsonism (DIP), tardive dyskinesia (TD), akathisia, myoclonus, and tremor, could occur in the treatment process of numerous psychiatric disorders. The symptoms appear days to months after taking the antipsychotics, particularly after the prescription of the first-generation antipsychotics (1). Antipsychotic drugs target the dopamine D2 receptors and show their therapeutic effect by blocking them. Inhibiting D2 receptors in the striatum ultimately results in a disturbance in thalamocortical circuitry (2). Old age, female gender, history of extrapyramidal symptoms, comorbidity disorders, and family history of Parkinson’s disease (PD) are listed as the risk factors for DIP occurrences (1).

Parkinsonism is a clinical diagnosis of a group of neurological disorders described by bradykinesia, rest tremor, and rigidity (3). There are many alternative causes to review before making an accurate diagnosis. The underlying causes could be neurodegenerative syndromes, such as dementia with Lewy bodies, multiple system atrophy, and PD (3, 4). Parkinson’s disease is a neurodegenerative disorder resulting from dopamine depletion in the striatum (5). Although the underlying mechanism has not been completely recognized yet, the most probable hypothesis is the accumulation of free radicals and oxidative stress (6). Drug-induced parkinsonism clinical presentations are sometimes described as symmetrical and bilateral, compared to PD symptoms (1). Experiencing these symptoms might suggest the patient’s diagnosis is DIP rather than the preclinical stages of PD. Since the core symptoms of PD and DIP are similar, distinguishing them is challenging. The most sensitive diagnostic tool to differentiate PD and DIP is dopamine transporter imaging with single-photon emission computed tomography (DaT-SPECT). Normal DaT-SPECT indicates DIP; however, in parkinsonism with neurodegenerative causes, such imaging method is abnormal (7).

The first step in managing DIP is to reduce the medication dose. If lowering the dose of responsible medication did not help resolve the symptoms or was not clinically possible, switching to a safer choice, such as second-generation antipsychotic medications, is an alternative approach. Finally, discontinuing the medication is suggested (1). Since it is usually impossible to discontinue the agent, prescribing an additive medication is essential. Currently, anticholinergic drugs and amantadine are used for the management of these symptoms (3).

Despite their beneficial results, there are serious concerns for the elderly using anticholinergics. They can cause adverse effects, such as urinary retention and cognitive impairment, and there is also a higher risk of developing TD. Amantadine might be used as an alternative treatment (8). Trials have indicated the effectiveness of amantadine in the case of patients suffering from anticholinergic side effects. Amantadine could stimulate the central nervous system resulting in several symptoms, such as psychosis, agitation, and delirium. These conditions could deem irritating for the patient to the point of discontinuing these medications (9).

*Ginkgo biloba* has shown promising results in animal models with parkinsonism to recover dopamine levels (6). Taking *Ginkgo biloba* extract (EGb) with a 240 mg/day dose is considered to be salutary (10). *Ginkgo biloba* extract is a monoamine oxidase B inhibitor and possesses antioxidant, anti-inflammatory, and anti-platelet activating factor (anti-PAF) features. The anti-PAF mechanism also increases cerebral blood flow (11). There is supporting evidence for using EGb as an adjunctive treatment in chronic schizophrenia and Alzheimer’s disease (12). It is also commonly used in other dementia syndromes, cerebrovascular events, and peripheral vascular disturbances (10, 11).

## 2. Objectives

The current study aimed to evaluate the safety and efficacy of EGb in relieving DIP symptoms. For this purpose, a randomized, double-blinded, placebo-controlled clinical trial was conducted in Roozbeh hospital in Tehran. Participants were selected from hospitalized psychiatric patients using antipsychotics and presenting with DIP manifestations.

## 3. Methods

### 3.1. Inclusion and Exclusion Criteria

All the patients with a history of psychiatric disorders who were over the age of 18 years and suspected to have any symptoms of parkinsonism were consulted by a neurologist. Drug-induced parkinsonism has been confirmed based on clinical judgments. All the patients with a history of diagnosed PD or other types of neurodegenerative parkinsonism were excluded from the study. In addition, a history of other neurological disorders consisting of stroke, dementia, and epilepsy was the definite exclusion criterion.

After the patient's comprehensive examination and application of the neuroimaging with Brain MRI, the evaluation of probable vascular and treatable causes of parkinsonism, and the adjustment of the related medication dosage, the diagnosis of probable DIP was made by a consultant neurologist.

At this stage, if the patient fulfilled the criteria, informed consent was obtained.

The study was registered in the Iranian Registry of Clinical Trials (Registration code: IRCT20200708048060N1).

### 3.2. Random Allocation

The patients were randomly assigned to the intervention and control groups using the block randomization method (with four blocks). To hide the allocation and maintain blindness, one of the researchers outside the field prepared a random sequence of patients and encoded each patient’s medication package. Drugs and placebo tablets, which were identical in appearance, were poured into similar cans. All tablets were packed in coded cans with a three-digit code. The patient list was also provided to the field-based researcher based on these three-digit codes. It was also worth mentioning that all clinical evaluations were performed by a neurologist, who was not involved in the allocation process.

### 3.3. Intervention

In the intervention group, the patients were treated with 80 mg of *Ginkgo biloba* pills (containing dry EGb) prescribed three times a day. In the control group, routine treatment with a placebo continued. Moreover, treatment with *Ginkgo biloba* or placebo pills continued for 3 months.

### 3.4. Characteristics of Data Collection Tools and Collection Method

The information collection form included patient demographic information (i.e., age, gender, and educational level), type and status of psychiatric illness, and the history of medications. The severity of parkinsonism symptoms was measured by the Unified Parkinson’s Disease Rating Scale (UPDRS).

There were no definite criteria or clear guidelines to assess DIP severity and symptoms. Numerous studies utilized different methods and batteries to assess the severity of parkinsonism in DIP, including Simpson Angus Scale (SAS), UPDRS, and other questionnaires. However, the UPDRS, especially the motor part of the UPDRS, is the most comprehensive questionnaire to evaluate Parkinsonism symptoms. On the other hand, DIP can be clinically indistinguishable from other types of neurodegenerative parkinsonism. As a result, the UPDRS could be applied to both disorders to assess the motor symptoms of parkinsonism. The UPDRS developed as a tool to measure Parkinson's disability, examines four areas, including nonmotor symptoms, the patient's ability to perform daily activities, the motor features of speech, facial expression, tremor, tone, movement slowness in the hands and legs, walking, and balance and ultimately measures the complications of treatment.

In this study, only the items of the movement examination section were asked.

Several studies have demonstrated cognitive difficulties in patients with different types of psychiatric disorders, including mood and psychotic disorders. These cognitive impairments frequently involve the domains of executive function, processing speed, and complex attention, which could be evaluated appropriately with the Montreal Cognitive Assessment (MoCA) battery. The Montreal Cognitive Assessment tool is used to assess cognition in these patients. This tool contains 30 items that take about 10 minutes to complete. A higher score indicates a better cognition status.

All the tests were evaluated two times, including the time of enrolment and 3 months after starting the medication.

### 3.5. Statistical Description and Data Analysis

Since all the questionnaires were completed two times for each person, repeated measures analysis was used for in-group and inter-group comparisons. If the necessary assumptions for this analysis were not provided, generalized estimating equation analysis would be replaced. These analyses can control the confounders and the base value within each group.

### 3.6. Ethical Considerations

Written informed consent was obtained from all the participants. The study was conducted in accordance with the Declaration of Helsinki 1964, its later amendments, and national legislation and institutional guidelines. The study protocol was approved by the Local Medical Ethics Committee of Tehran University of Medical Sciences, Tehran, Iran (IR.TUMS.TIPS.REC.1398.097).

(webpage of the ethical approval code: http://ethics.research.ac.ir/IR.TUMS.TIPS.REC.1398.119)

## 4. Results

As previously mentioned, the purpose of this clinical trial was to evaluate the effectiveness of *Ginkgo biloba* in DIP in patients admitted to the Psychiatric Ward of Roozbeh hospital. The finalized data of 63 patients were included in the study. After mentioning the demographic and the descriptive information of each variable, the specific objectives of the study were examined.

In the experimental group, there was an equal number of female (n = 16, 50%) and male (n = 16, 50%) patients, with a mean age of 51.75 years. In the control group, 18 (58%) and 13 (41.9%) patients were female and male, respectively, with a mean age of 48.7 years. In the experimental group, 62.1% of patients were diagnosed with mood disorders, including bipolar mood disorder and major depressive disorder. Nevertheless, in the control group, 65% of patients were diagnosed with mood disorders. Psychotic disorders, including schizophrenia and schizoaffective disorders, accounted for about 33.3% of patients in the experimental group. Nonetheless, in the control group, 25% of patients were diagnosed with psychotic disorders (Table 1).

**Table 1.** Demographic Data of Study Groups ^a^

	Control Group	Experimental Group	P-Value	Statistics
**Gender**			0.521	χ^2^ = 0.412
Female	18 (58)	16 (50)		
Male	13 (41.9)	16 (50)		
**Psychiatric disorders**			0.908	χ^2^ = 1.542
Schizophrenia	2 (10.0)	2 (11.1)		
Bipolar mood disorder	8 (40.0)	8 (44.4)		
Major depressive disorder	5 (25.0)	3 (16.7)		
Schizoaffective	3 (15.0)	4 (22.2)		
Obsessive-compulsive disorder	1 (5.0)	1 (5.6)		
Autism spectrum disorder	1 (5.0)	0 (0)		
Non-confirmed diagnosis	11	14		
Total	31 (100)	32 (32)		
**Age, y**	48.70 ± 14.53	51.75 ± 14.25	0.408	*t* = 0.834

^a^ Values are expressed as No. (%) or mean ± SD.

### 4.1. Determining the Effectiveness of Ginkgo biloba in the Improvement of Patients’ Parkinsonism Symptoms

The difference between the experimental and control groups in each of the components of the UPDRS under the influence of *Ginkgo* was statistically confirmed, shown in Table 2 and 3.

**Table 2.** Double Comparison of Research Groups in Post-test Scores of Unified Parkinson’s Disease Rating Scale Components

Dependent Variable	(I) Group	(J) Group	Mean Difference (I-J)	Standard Error	P-Value
**Tremor severity 2**	Experimental	Control	-4.952*	1.013	0.000
**Motor symptoms severity 2**	Experimental	Control	-7.438*	1.514	0.000
**Rigidity severity 2**	Experimental	Control	-0.400*	0.189	0.039
**Bradykinesia severity 2**	Experimental	Control	-2.198*	0.453	0.000

**Table 3.** Analysis of Covariance to Evaluate the Effectiveness of *Ginkgo biloba* on Unified Parkinson’s Disease Rating Scale Components

Source	Dependent Variable	Type III Sum of Squares	df	Mean Square	F	P-Value
**Tremor severity 1**	Tremor severity 2	17.227	1	17.227	1.220	0.274
**Motor symptoms severity 1**	Motor symptoms severity 2	316.488	1	316.488	10.024	0.003
**Rigidity severity 1**	Rigidity severity 2	5.043	1	5.043	10.281	0.002
**Bradykinesia severity 1**	Bradykinesia severity 2	8.645	1	8.645	3.062	0.086
**Group**	Tremor severity 2	337.575	1	337.575	23.904	0.000
	Motor symptoms severity 2	761.703	1	761.703	24.126	0.000
	Rigidity severity 2	2.201	1	2.201	4.487	0.039
	Bradykinesia severity 2	66.540	1	66.540	23.565	0.000

The severity of DIP motor symptoms (based on the UPDRS score) in patients after the intervention was reduced from 17.97 to 11.83 in the *Ginkgo biloba* group, compared to 14.27 in the placebo group, which was increased to 15.60. The rate of change in the severity of the tremor decreased in the *Ginkgo biloba* group from 9.56 before the intervention to 6.03 after that. However, in the control group, the tremor intensity was 7.30 at the baseline, which increased to 8.90 after receiving the placebo. Therefore, *Ginkgo* had a high probability of reducing the severity of tremor symptoms (F = 23.904, P < 0.0001).

The rate of change in the severity of rigidity symptoms in the *Ginkgo biloba* group decreased from 1.53 before the intervention to 1.20 after that. Nonetheless, in the control group, the stiffness intensity was 1.37, which increased to 1.40 after receiving the placebo. Consequently, *Ginkgo* was proven to lead to the improvement of rigidity (F = 4.487, P < 0.0001).

The rate of change in the severity of bradykinesia symptoms decreased from 5.5 before the intervention to 3.67 after the intervention in the group receiving *Ginkgo biloba*. However, in the control group, the severity of bradykinesia was 4.43, which increased to 4.80 upon receiving the placebo. *Ginkgo* extract could result in a change in the severity of bradykinesia (F = 23.565, P < 0.0001)

### 4.2. Determining the Effectiveness of Ginkgo biloba in the Improvement of Patients’ Cognition (Based on MoCA Score)

The MoCA score in the group of patients receiving *Ginkgo biloba* increased from 19.28 to 21.38 after receiving the medication. Nevertheless, in the control group, the cognitive evaluation score did not change significantly before and after receiving the placebo (20.48 and 20.60 before and after the placebo, respectively).

Working memory in the group of patients receiving *Ginkgo* increased from 3.66 to 4.21 afterward. Nonetheless, in the placebo group, the score of the working memory test subsequently did not have any significant difference (3.80 and 3.83 before and after receiving the placebo, respectively). The recent memory score in the group of patients receiving *Ginkgo* increased from 1.81 to 2.62. However, in the placebo group, the recent memory score dropped from 2.20 to 2.13.

The verbal test in the *Ginkgo* group increased from 4.56 to 4.86 afterward. Nevertheless, in the placebo group, the score before and after the placebo did not differ significantly (4.70 and 4.80 before and after receiving the placebo, respectively). However, the difference in the intervention group was not statistically significant.

There was no significant difference in the orientation component of the MoCA test in the *Ginkgo* group (4.25 and 4.24 before and after, respectively). In addition, in the placebo group, the score of the orientation test before and after the placebo was slightly different. Tables 4 and 5 show the binary comparisons of the pre-test and post-test scores of the two groups and the differences in scores.

**Table 4.** Double Comparison of Research Groups in Post-test Scores of Montreal Cognitive Assessment Components

Dependent Variable	(I) Group	(J) Group	Mean Difference (I-J)	Standard Error	P-Value
**Working memory 2**	Experimental	Control	0.460*	0.213	0.036
**Language 2**	Experimental	Control	0.019	0.136	0.892
**Executive function 2**	Experimental	Control	0.191	0.167	0.258
**Short memory 2**	Experimental	Control	0.834*	0.290	0.006
**Abstract 2**	Experimental	Control	0.025	0.103	0.807
**Orientation 2**	Experimental	Control	0.090	0.208	0.669

**Table 5.** Analysis of Covariance Test for the Effectiveness of *Ginkgo biloba* in Montreal Cognitive Assessment Components

Sources	Dependent Variable	Type III Sum of Squares	df	Mean Square	F	P-Value
**Working memory 1**	Working memory 2	54.733	1	54.733	88.839	0.000
**Language 1**	Language 2	14.012	1	14.012	56.234	0.000
**Executive function 1**	Executive function 2	75.800	1	75.800	201.579	0.000
**Short memory 1**	Short memory 2	18.540	1	18.540	16.290	0.000
**Abstract 1**	Abstract 2	11.172	1	11.172	78.002	0.000
**Orientation to time and place 1**	Orientation to time and place 2	22.172	1	22.172	37.732	0.000
**Group**	Working memory 2	2.860	1	2.860	4.643	0.036
	Language 2	0.005	1	0.005	0.019	0.892
	Executive function 2	0.492	1	0.492	1.309	0.258
	Short memory 2	9.398	1	9.398	8.258	0.006
	Abstract 2	0.009	1	0.009	0.060	0.807
	Orientation to time and place 2	0.108	1	0.108	0.185	0.669

### 4.3. Clinical Complications and Side Effects

The types of side effects (in the drug and placebo groups) were observed during the trial. The difference between the drug and placebo in the frequency of side effects was not significant (Table 6).

**Table 6.** Number of Patients with Side Effects ^a^

Side Effect Type	Drug Group	Placebo Group
**Headache**	1 (0.030)	1 (0.032)
**Nausea**	2 (0.060)	1 (0.032)
**Dizziness**	2 (0.060)	1 (0.032)
**Skin allergic reaction**	1 (0.030)	0 (0)

^a^ Values are expressed as No. (%).

Only one serious complication was observed during the clinical trial conducted on the patients. After participating in this study, one of the patients expressed that he had skin rashes, due to which he was excluded from the study. Several cases of mild gastrointestinal side effects, headache, and dizziness were also observed, which did not require intervention.

## 5. Discussion

As previously mentioned, *Ginkgo biloba* could lead to a change in the intensity of rest tremor (F = 23.904, P < 0.0001), the severity of motor symptoms (F = 24.126, P < 0.0001), rigidity (F = 4.487, P < 0.0001), and bradykinesia (F = 23.565, P < 0.0001). *Ginkgo biloba* leads to a change in the cognitive function of the two domains, including working memory (F = 4.643, P = 0.036) and recent memory (F = 8.258, P = 0.006). However, the difference between the two groups in other cognitive domains was not significant.

A review of other studies, namely a meta-analysis by Zheng et al., suggests the effectiveness of EGb in the treatment of TD in patients with schizophrenia. In this study, TD severity was measured based on the Abnormal Involuntary Movement Scale (AIMS), and drug side effects were evaluated based on Treatment Emergent Symptom Scale. This study demonstrated that EGb could improve the TD symptoms based on the AIMS compared to the control group. The possible mechanism of EGb in the treatment of TD is trapping the free radicals directly and preventing the formation of free radicals indirectly, which reduces oxidative stress and improves the brain-derived neurotrophic factor (BDNF) level (13).

Another study was conducted at Shahid Beheshti University, Tehran, Iran, in 2015 on the *Ginkgo biloba* effect on the improvement of oropharyngeal dyskinesia. The aforementioned study demonstrated that the concomitant prescription of EGb can improve behavioral and biochemical changes as a result of reducing oxidative stress following the long-term use of neuroleptic drugs. The long-term use of haloperidol (1 mg/kg) significantly increased vacuous chewing movements (VCMs). The concomitant use of *Ginkgo* at a dose of 25 mg/kg with haloperidol reduces VCMs (2).

A 2013 study by the Brazilian Department of Psychiatry examined the effects of *Ginkgo biloba* on an animal model of PD. One of the possible causes of PD is oxidative stress and the production of active oxygen in the substantia nigra. The hypothesis proposed in the aforementioned study was the neuroprotective effects of EGb versus 6-hydroxydopamine, 1-methyl1-4-phenyl-1,2,3,6-tetrahydropyridine, and MPP toxins (14).

In a study evaluating the dose-dependent effect of *Ginkgo biloba* against 6-hydroxy dopamine-induced parkinsonism in an animal model, mice were treated with three doses of 50, 100, and 150 mg/kg for 3 weeks. The 6-OHDA injection was given, and 3 weeks later, changes in locomotor activity and muscle coordination, increased thiobarbituric acid reactive substances, and a dramatic decrease in glutathione concentration in the substantia nigra were evaluated. The findings indicated that these variables were improved by EGb. *Ginkgo biloba* extract increases Tyrosine Hydroxylase-ImmunoReactive (TH-IR) fibers in the ipsilateral substantia nigra. In general, EGb can be used as a treatment to reduce the effects of neuronal damage in parkinsonism in animal models (11).

Another study was conducted in Mexico in 2015 on the use of *Ginkgo* in psychiatric diseases. The possible mechanisms of positive effects of EGb761 on psychiatric disorders, such as depression, anxiety disorders, and schizophrenia, could include the antioxidant effect, the modulation effect on neurotransmitters, the regulation of neuroendocrine systems, and the level of neurotrophic factors. On the other hand, it has minimal side effects, including gastrointestinal complications and an increased risk of bleeding. In dementia syndromes, this compound improves the patient’s cognitive function, psychological symptoms, daily activities, and quality of life. The safety, tolerability, and efficacy of a daily dose of 240 mg have been evaluated in many clinical trials (10).

In the meta-analysis of 8 double-blinded, randomized, and placebo-controlled studies in China, the effect of EGb was evaluated as adjunctive therapy in chronic schizophrenia on 1033 patients. The results showed a significant difference between the two groups in improving the overall symptoms and negative symptoms of chronic schizophrenia in favor of the EGb group. Additionally, antipsychotic treatment is more effective than EGb.

After reviewing all the studies to determine the effectiveness of EGb in psychiatric and neurological diseases, to date, no study has been performed to evaluate the effectiveness of EGb in DIP and its associated cognitive decline in psychiatric patients. The present study is the first investigation to evaluate the effectiveness of *Ginkgo biloba* in this field.

According to the positive results of this study regarding the improvement of motor symptoms in general and the improvement of resting tremor intensity, bradykinesia, and rigidity as the primary and direct result of this study, the findings also revealed the positive effect on cognition in patients with psychiatric disorders, including psychotic disorders and mood disorders, with the most positive cognitive effect on working memory and recent memory. The improvement of cognitive status and especially recent memory and working memory of these patients as a secondary result of this study provides a good perspective on drug use in future studies, specifically in improving cognitive disorders in psychiatric patients with mood and psychotic disorders separately.

On the other hand, with the improvement of memory status and the complications of DIP in these patients, the executive and job performance of patients and, in general, the patient’s quality of life will be significantly improved. Therefore, in future studies, the quality of life of patients should be considered a secondary goal of studies.

### 5.1. Study Limitations

In this study, there are some limitations that can be pointed out and considered in future studies to provide stronger evidence in favor of using EGb. Firstly, all patients admitted to psychiatric wards were included in the study.

This study evaluated different categories of psychiatric disorders, including schizophrenia, schizoaffective disorder, bipolar disorder, depression, and even psychiatric diseases, such as obsessive-compulsive disorder. The study groups could be more homogeneous and obtain results with more evidence.

On the other hand, due to the coronavirus disease 2019 (COVID-19) pandemic, fewer patients with a higher probability of severe psychiatric illness entered the study, which can affect the results. Therefore, subsequent studies with a higher number of patients after the COVID-19 pandemic can be more effective. This study included psychiatric patients with more access and more likely follow-up. In future studies, it is recommended to include patients in clinics and outpatients.

All patients with DIP are initially treated with anticholinergics or amantadine. Therefore, it is necessary to carefully record the type of medications and dosage as confounding factors in the analysis.

Dopamine Transporter Scan (DaT scan), Dopamine Transporter Scan (DaT scan) is broadly utilized for the differential diagnosis of PD and other degenerative parkinsonism from drug-induced parkinsonism, which is not routinely performed on the current study’s patients. Therefore, in a small percentage of patients, the symptoms might be due to PD, which is unmasked by antipsychotic drugs.

### 5.2. Conclusions

This study is valuable given that patients with DIP and neurodegeneration can both benefit from EGb despite the above-mentioned limitations. It is proposed that future studies confirm their results with a DaT scan. Moreover, it is recommended to evaluate patients’ quality of life and cognitive status with more comprehensive cognitive tests.

## Clinical Trial Registration Code:

The study was registered in the Iranian Registry of Clinical Trials (Registration code: IRCT20200708048060N1).
